# Regulatory T cells modulate inflammation and reduce infarct volume in experimental brain ischaemia

**DOI:** 10.1111/jcmm.12304

**Published:** 2014-05-30

**Authors:** David Brea, Jesús Agulla, Manuel Rodríguez-Yáñez, David Barral, Pedro Ramos-Cabrer, Francisco Campos, Angeles Almeida, Antoni Dávalos, José Castillo

**Affiliations:** a Department of Neurology, Neurovascular Area, Clinical Neurosciences Research Laboratory, Hospital Clínico Universitario, Health Research Institute of Santiago de Compostela (IDIS), Universidade de Santiago de CompostelaSantiago de Compostela, Spain; b Cellular and Molecular Neurobiology Research Group and Grup de Recerca en Neurociencies del IGTP, Department of Neurosciences, Fundació Institut d'Investigació en Ciències de la Salut Germans Trias I Pujol-Universitat Autónoma de BarcelonaBadalona, Spain; c Instituto de Investigación Biomédica de Salamanca, Hospital Universitario de SalamancaSalamanca, Spain

**Keywords:** cerebrovascular disease/stroke, regulatory T cells, inflammation, immunomodulation

## Abstract

Brain ischaemia (stroke) triggers an intense inflammatory response predominately mediated by the accumulation of inflammatory cells and mediators in the ischaemic brain. In this context, regulatory T (Treg) cells, a subpopulation of CD4^+^ T cells with immunosuppressive and anti-inflammatory properties, are activated in the late stages of the disease. To date, the potential therapeutic usefulness of Treg cells has not been tested. In this study, we aimed to investigate whether Treg cells exert protection/repair following stroke. Both the adoptive transfer of Treg cells into ischaemic rats and the stimulation of endogenous T-cell proliferation using a CD28 superagonist reduced the infarct size at 3–28 days following the ischaemic insult. Moreover, T cell-treated animals had higher levels of FoxP3 and lower levels of IL-1β, CD11b+ and CD68+ cells in the infarcted hemisphere when compared with control animals. However, T-cell treatment did not alter the rate of proliferation of NeuN-, NCAM- or CD31-positive cells, thereby ruling out neurogenesis and angiogenesis in protection. These results suggest that adoptive transfer of T cells is a promising therapeutic strategy against the neurological consequences of stroke.

## Introduction

Regulatory T cells (Treg cells) are a subpopulation of CD4^+^ T cells that play a role in maintaining immune homoeostasis, preventing autoimmunity and inflammation. Treg cells are also responsible for limiting tissue damage during immune/inflammatory responses [1].

Following ischaemic stroke, cellular death by necrosis activates an intense inflammatory response [2], which is characterized by the accumulation of inflammatory cells and mediators in the ischaemic brain and systemic changes in inflammatory cells, cytokines and chemokines [3]. Evidence suggests that inflammation contributes to tissue injury after stroke. Thus, it has been shown that defects in the immune system, such as a genetic deficiency of adhesion molecules or immunodeficiency, attenuate brain damage after ischaemia [3]. Furthermore, white blood cell counts and levels of C-reactive protein, serum/plasma interleukin-6 (IL-6) and tumour necrosis factor-α (TNF-α) are increased in stroke patients [4]; several of these factors have been associated with stroke outcome [5] and recurrence [6].

Although it is known that immune responses contribute to tissue damage, attempts to improve stroke outcome by immunosuppression strategies have been so far unsuccessful [7]. However, immunosuppressive mechanisms that involve Treg cells may represent an effective strategy because Treg cells are natural agents that do not suppress, but rather, control immune responses under physiological or pathological conditions. Indeed, Treg cells have been successfully used to reduce damage in ischaemic kidney injury [8] and liver ischaemia–reperfusion injury [9].

In the context of brain ischaemia, it has been demonstrated that Treg-cell levels increase after ischaemia [10,11], and that mucosal tolerance to E-selectin (a neuroprotectant) is mediated by Treg cells [12]. However, the role of Treg cells in cerebral ischaemia remains controversial. Thus, Liesz *et al*. [13] showed that animals subjected to Treg-cell depletion presented larger infarct volumes, contradicting the studies of Ren *et al*. [14], who used a different method to deplete Treg cells and found no effects on infarct volumes. In addition, Kleinschnitz *et al*. [15] showed that Treg cells exacerbated brain injury early after transient ischaemia, while others described that adoptive regulatory T-cell therapy protected against cerebral ischaemia [16]. Finally, some authors have shown that Treg cells impair [17], whereas others claim that they improve [18], angiogenesis after limb ischaemia, but little is known about the effects of Treg cells in angiogenesis or neurogenesis after cerebral ischaemia. For these reasons, it has been generally agreed that further studies are needed to clarify the role of Treg cells in cerebral ischaemia [15,19,20].

In this work, we studied the role of Treg cells in brain ischaemia using two different strategies (*i.e*., adoptive transfer of exogenous Treg cells and stimulation of endogenous Treg cells); we analysed the long-term effects of regulatory T cells after ischaemia with special attention focused on their effects on neuroblasts division and angiogenesis.

## Materials and methods

### Purification and *in vitro* expansion of regulatory T cells

Single-cell suspensions were obtained from the neck and mesenteric lymph nodes of Sprague-Dawley male rats and incubated with FITC-conjugated anti-CD4 and PE-conjugated anti-CD25 antibodies (BD Biosciences, Franklin Lakes, NJ, USA), both at 10 μl/10^6^ cells. CD4^+^CD25^+^ (regulatory T cells; Treg) and CD4^+^CD25^−^ cells (conventional T cells; Tconv) were sorted by using a FACSAria I cell sorter (BD Biosciences), achieving 84.7 ± 4.8% and 97.4 ± 0.9% of purity respectively. Fresh Tconv cells were used in co-cultures with Treg cells for *in vitro* suppression assays, whereas Treg cells were expanded *in vitro*, as previously described [21].

### *In vitro* suppression assays

*In vitro* suppression assays were performed to verify the immunosuppressing properties of expanded Treg cells. Freshly isolated Tconv cells were stained with carboxyfluorescein succinimidyl ester diacetate (CFSE) dye (5 μM; Invitrogen, Carlsbad, CA, USA) and 5 × 10^4^ cells per well (24-well plates) were stimulated to proliferate with 2 μg/ml of anti-TCR mAb (BD biosciences) and 10 μg/ml of anti-CD28 mAb (clone JJ316; BD Biosciences). To determine the immunosuppressing capacities of Treg cells, different numbers of Treg cells were added (0, 1.25 × 10^4^, 2.5 × 10^4^ and 5 × 10^4^). Tconv and Treg cells were co-cultured for 3 days, when cells were analysed by flow cytometry. Proliferation was measured by determining the dilution of CFSE fluorescence [22].

### Brain ischaemia rat model

Transient focal cerebral ischaemia was induced in Sprague-Dawley male rats by intraluminal occlusion of the middle cerebral artery (tMCAO), performed as previously described [23]. Only rats with a ≥75% reduction in hemispheric cerebral blood flow (measure by laser Doppler flow) were included in the study.

All procedures were performed under EU regulations (European Communities Council Directive of 24 November 1986 - 86/609/EEC, 2003/65/CE, 2010/63/EU), with the approval of our institution's ethics committee.

### Treatment groups

In a first set of experiments, we assessed the neuroprotective role of the adoptive transfer of Treg cells. Twenty rats were randomized between a control (*n* = 10) and a Treg-treated group (*n* = 10). Controls received an i.v. injection of 1 ml of PBS, 2 hrs after the induction of ischaemia, whereas Treg-treated rats received an injection of 3 × 10^6^ expanded Treg cells in 1 ml of PBS. Infarct volumes were analysed by magnetic resonance imaging (MRI) at days 1, 3, 7 and 10. At day 10, animals were sacrificed and the brains were used to analyse IL1β, FoxP3, CD11b and CD68 positive cells.

In the second set of experiments, rats received an i.v. injection of 1 ml of PBS (*n* = 8), or 500 mg of anti-CD28 mAb [24] (clone JJ316; BD Biosciences) in 1 ml of PBS (*n* = 8), 4 days before the ischaemia. Infarct volumes were analysed by MRI at days 1, 3, 7 and 10.

In a final set of experiments, rats were randomized between a control group (*n* = 8) that received an i.v. injection of 1 ml of PBS, two hours after the induction of ischaemia, and a Treg-treated group (*n* = 8) that received an injection of 3 × 10^6^ expanded Treg cells in 1 ml of PBS. Infarct volumes were analysed by MRI at days 7, 14, 21 and 28. Between days 7 and 21, all animals received an i.p. injection of BrdU (Sigma-Aldrich, Buchs SG, Switzerland; 50 mg/Kg). At day 29, animals were sacrificed and the brains were used to analyse BrdU+, NeuN+, BrdU+NCAM+ and BrdU+CD31+ cells.

### MRI analysis

Magnetic resonance images were acquired at 9.4 Tesla (Bruker Biospec, Ettlingen, Germany). T2-weighted images were obtained by using RARE (Rapid Acquisition with Refocused Echoes) pulse sequences with a train of *n* = 4 echoes. Two imaging settings were used with the following parameters: (1) Effective echo time TE_eff_ = 45 ms, repetition time TR = 3.5 sec., signal average *n* = 2, field-of-view FOV = 19.2 × 19.2 mm^2^, matrix 192 × 192 points, in-plane resolution of 0.1 × 0.1 mm^2^, 14 consecutive slices of 1 mm thickness and an acquisition time of 5 min. 36 sec. (2) Effective echo time TE_eff_ = 60 ms, repetition time TR = 2.0 sec., signal average *n* = 1, field-of-view FOV = 28.8 × 28.8 mm, matrix 192 × 192 points, in-plane resolution of 0.15 × 0.15 mm^2^, 14 consecutive slices of 1 mm thickness and an acquisition time of 1 min. 12 sec. Images were analysed and processed by a scientist blinded to the study by using Bruker's Paravision 5.1 software and Image-J (Rasband, W.S., ImageJ, U. S. National Institutes of Health, Bethesda, MD, USA, http://imagej.nih.gov/ij/, 1997–2011).

After measuring the volume of both brain hemispheres, oedema formation was quantified by the difference in hemispheric volumes by using the following equation: Oedema (%) = 100*[1 − (V1/V2)], with V1 and V2 representing the contralateral and ipsilateral hemisphere volumes, respectively.

### Western blot and ELISA analyses

Western blot analyses were performed to quantify FoxP3 expression (Treg marker), and ELISA tests were performed to quantify interleukin-1β (IL-1β) in the rat brain following ischaemia at day 10. Brains were extracted, and the infarcted and contralateral hemispheres were separated and sonicated with RIPA buffer (Sigma-Aldrich, St. Gallen, Switzerland) and centrifuged at 18,000 × g for 30 min. at 4°C. Protein concentrations of the supernatants were calculated by using the Bradford method.

Western blots were performed by using 50 μg of protein, separated with 10% SDS-PAGE and blotted onto PVDF membranes (Millipore, Billerica, MA, USA). The membranes were blocked with 5% fat-free dehydrated milk in TBS (Tris Buffered Saline, Sigma-Aldrich) for 2 hrs and incubated overnight at 4°C with mouse anti-rat FoxP3 (1:75 dilution; Santa Cruz Biotechnology, Heidelberg, Germany) and rabbit anti-rat actin (1:3000; Abcam, Cambridge, United Kingdom). The membranes were incubated with goat antimouse IgG-Cy3 and goat anti-rabbit IgG-Cy5 (1:3000 both; GE Lifesciences, Munich, Germany) for 2 hrs and were digitized by using a FXProplus scanner (Bio-Rad, Hercules, CA, USA). The intensity of bands was analysed with Quantity One software (Bio-Rad).

ELISAs for IL-1β was acquired from GE Lifesciences, and the quantification of these proteins was performed according to the manufacturer's instructions.

### Flow cytometry determination of neuroblasts division and angiogenesis

Brain hemispheres were separated and homogenized by using a cell strainer. Cells were washed and stained by incubation (15 min.) with NCAM-PE (10 μl per 10^6^ cells; Miltenyi Biotec, Bergisch Gladbach, Germany) or CD31-PE (2 μl per 10^6^ cells; BD Biosciences, San Jose, CA, USA) antibodies. Then, cells were suspended in 100 μl of Cytofix-cytoperm (BD Biosciences) and stained for BrdU by using an APC BrdU Flow Kit from BD Pharmigen (BD Biosciences), according to the manufacturer's instructions. Finally, cells were stained with NeuN-Alexa Fluor 488 (10 μl per 10^6^ cells; Chemicon Millipore, Temecula, CA, USA), and 50,000 cell events were acquired in a BD FACS Aria I (BD Biosciences); the number of double-positive NeuN-BrdU, NCAM-BrdU or CD31-BrdU cells were counted [25].

### Immunohistochemistry for CD11b and CD68

Immunohistochemistry (IHC) of inflammatory cells was performed on three animals per group. Extracted brains were cut into 3-mm thick coronal slices and immersed in a 20% sucrose solution for 2 hrs at 4°C. Brain slices were embedded in OCT (Sakura Finetek Inc., Torrance, CA, USA) and stored at −80°C. For immunostaining, 10 μm coronal sections were obtained by using a cryostat (Tissue-Tek Cryo3; Sakura Finetek Inc.). Sections were further incubated with 3% H_2_O_2_ and 10% methanol in PBS, to block endogenous peroxidase. Sections were subsequently incubated overnight at 4°C with primary antibodies against CD68 (ED-1, 1:50 dilution; Abcam) and CD11b (OX42, 1:50 dilution; Abcam), followed by incubation with a biotin-conjugated secondary anti-rabbit antibody (1:200; Vector Laboratories Inc., Burlingame, CA, USA) for 1 hr and streptavidin-conjugated peroxidase (Vecstatin Abc kit, Vector Laboratories Inc.) for 30 min. The slices were developed by incubation (3 min.) with DAB 0.05% (w/v; Dako, Glostrup, Denmark) and visualized under an IX-51 microscope (Olympus Life and Material Science Europe GMBH, Hamburg, Germany), which was attached to a DS-U2 LCD camera (Nikon Instruments Inc., Melville, NY, USA). All slides were evaluated by a blinded, trained observer. Cells were counted in five representative fields of 192 μm^2^ in the infarcted area.

### Statistical analysis

Unless otherwise indicated, results are expressed as the mean ± SEM, by using Student's *t*-tests to compare variables between groups. Values of *P* < 0.05 were considered statistically significant. Statistical analysis was conducted by using SPSS 16.0 (SPSS, IBM Corporation, Somers, NY, USA) for Windows XP.

## Results

### Cultured Tregs reduce in vitro proliferation of T conventional cells

**Fig. 1:**
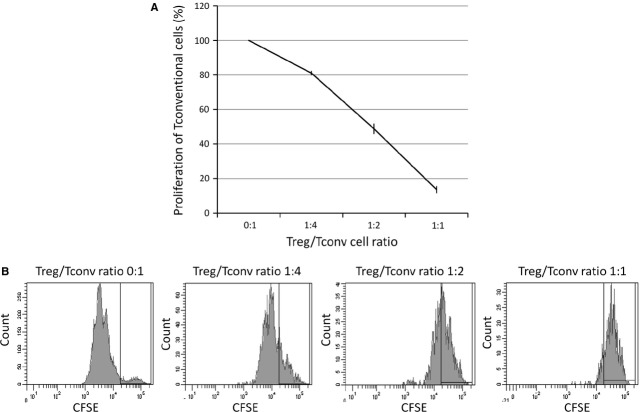
Suppression of the proliferation of conventional T cells by expanded regulatory T cells. (**A**) Regulatory T cells were isolated from SD rats, expanded for 5 days, serially diluted and added to Tconv cell cultures at different Treg/Tconv ratios as indicated. The percentage of proliferation of Tconv cells was determined by CFSE fluorescence. (**B**) Mono-parametric representation of CFSE fluorescence of Tconv cells co-cultured with Treg cells at the indicated ratios. High fluorescence is indicative of low proliferation (ratio 1:1), while low fluorescence is indicative of high proliferation (ratio 0:1). The black gate in the graphic indicates the position of non-proliferating cells. The experiment was repeated three times.

*In vitro* suppression assays combining fresh Tconv cells with expanded Treg cells were performed to verify the immunosuppressor properties of the expanded Treg cells. Treg cells were co-cultured for 3 days at a 1:1 ratio, achieving an 86.4 ± 3.0% suppression of Tconv cell proliferation (Fig. 1).

### Treg cells reduce infarct volume in rats after brain ischaemia

**Fig. 2:**
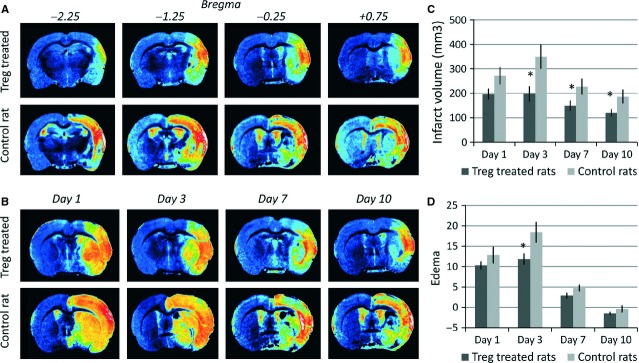
Temporal profiles of infarct and oedema, as measured by MRI, in control and Treg-treated animals. (**A**) T2 weighted MR images of four consecutive slices (position relative to bregma indicated in mm on top of the images) of the ischaemic brain of a control and a treated animal, acquired 10 days after induction of the infarct. (**B**) T2 weighted MR images of the same animals showing the temporal evolution of the lesion for control and treated animals. (**C**) Infarct volumes of Treg-treated (*n* = 10) and control rats (*n* = 10) at days 1, 3, 7 and 10 after ischaemia. (**D**) Oedema measured for control (*n* = 10) and Treg-treated rats (*n* = 10) at days 1, 3, 7 and 10 post MCAO. Columns represent mean values, and error bars represent SEM (* indicates statistically significant differences; *P* < 0.05).

Representative sets of four consecutive MRI brain slices from controls and a Treg-treated animal are presented in Figure 2A. A single MRI slice of the brain through time is presented in Figure 2B, depicting the divergent temporal evolution of infarct sizes observed in control and Treg-treated animals. Figure 2C presents the temporal evolution of infarct sizes, showing that Treg-treated animals displayed smaller lesions compared with controls at day 3 (198.01 ± 29.61 *versus* 349.84 ± 48.50 mm^2^; *P* = 0.027), day 7 (149.16 ± 19.43 *versus* 227.24 ± 30.64 mm^2^; *P* = 0.049) and day 10 after occlusion (120.38 ± 12.94 *versus* 186.71 ± 26.88 mm^2^; *P* = 0.041). In addition, rats treated with Treg cells also showed a reduction in brain oedema at day 3 following occlusion (Fig. 2D).

### Treg-treated animals present higher expression of FoxP3 and lower inflammatory mediators in the ischaemic hemisphere

**Fig. 3:**
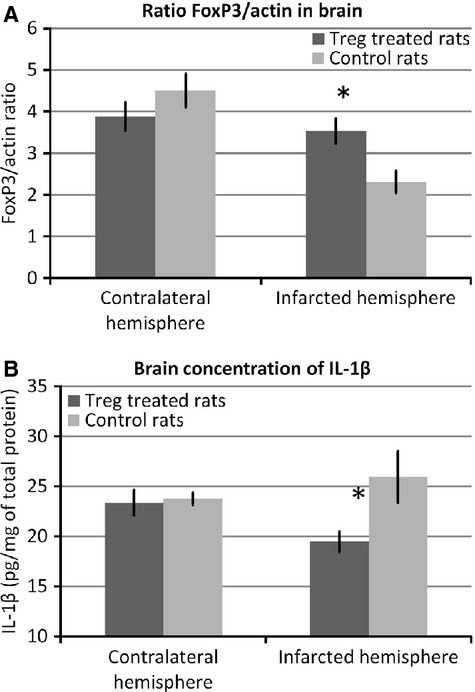
FoxP3 and inflammatory marker levels in rat brains. (**A**) FoxP3 expression in Treg-treated (*n* = 7) and control animals (*n* = 7), analysed for both the contralateral and infarcted hemispheres. FoxP3 was quantified from western blot images and normalized by actin intensity to eliminate small variations in loading between different samples. (**B**) Brain concentration of IL-1β in the contralateral and infarcted hemispheres of Treg-treated (*n* = 7) and control rats (*n* = 7), as determined by ELISA. In both graphs, columns represent mean values, and error bars represent SEM (* indicates statistically significant differences; *P* < 0.05).

Because Treg cells can infiltrate the ischaemic brain [11] (similar to lymphocytes), we analysed the expression of FoxP3 by western blotting in the contralateral *versus* the infarcted brain hemispheres. As shown in Figure 3A, FoxP3 expression in the infarcted hemisphere was higher for Treg-treated rats compared with controls (3.5 ± 0.3 *versus* 2.3 ± 0.3 AU; *P* = 0.015).

Because FoxP3 was overexpressed in Treg-treated rats, and considering that Treg cells are known immunosuppressing cells, we analysed the levels of the inflammatory marker IL-1β in both brain hemispheres. Levels of this marker were lower in the infarcted hemisphere of Treg-treated animals (19.46 ± 1.02 *versus* 25.92 ± 2.58 pg/mg of total protein; *P* = 0.048; Fig. 3B).

**Fig. 4:**
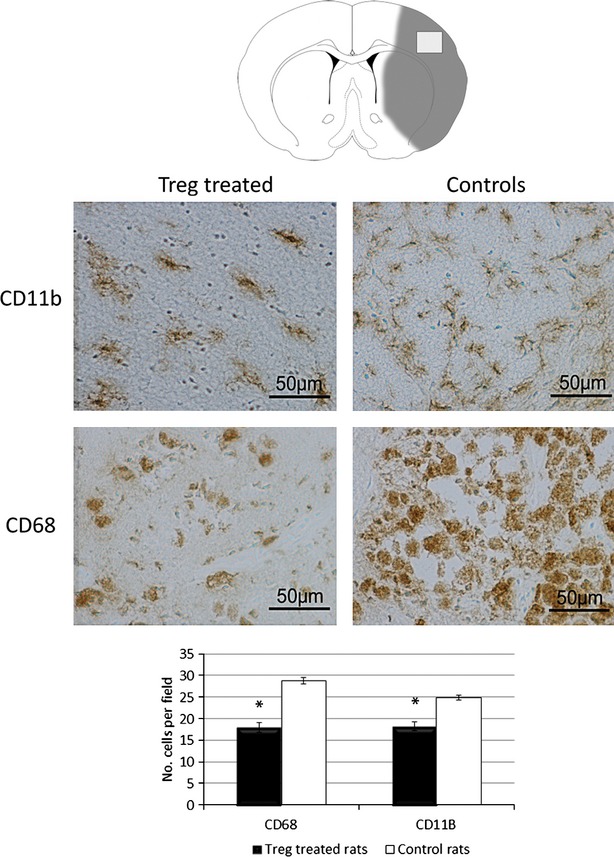
Analysis of CD68+ and CD11b+ cells in the brains of Treg-treated and control rats. Images and graph representation of immunohistochemistry staining for CD11b (first row) and CD68 (second row) for both treatment groups (*n* = 3 for each group). Treg-treated animals showed a marked decrease of both inflammatory markers at day 10. Pictures were taken at 40 × (bar 50 μm).

To study the influence of Treg treatment on the activity of inflammatory cells, we analysed the inflammatory cell markers in the infarct region of Treg-treated and control animals. Treg-treated animals exhibited lower numbers of CD68+ cells (*n* = 17.93 ± 1.07 *versus* 28.8 ± 0.7; *P* = 0.005) and CD11b+ cells (*n* = 18.23 ± 1.04 *versus* 24.85 ± 0.55; *P* = 0.018) compared with control animals (Fig. 4).

### Treatment with a CD28 superagonist also induces protection

**Fig. 5:**
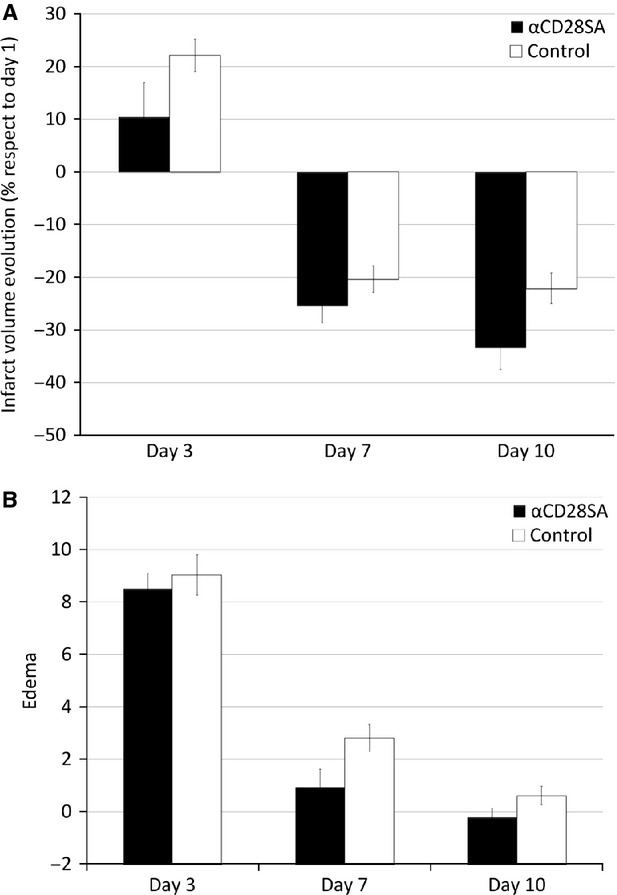
Temporal profiles of infarct and oedema, as measured by MRI, in control and CD28-SA-treated animals. (**A**) Infarct volume (expressed as the percentage of change from day 1) and oedema (**B**) progression with time for CD28-SA-treated (*n* = 8) and control animals (*n* = 8).

An additional experiment was performed to ensure the active role of Treg cells in protection after cerebral ischaemia. A group of animals were treated with CD28-SA (CD28 superagonist), which has been shown to induce endogenous Treg-cell proliferation [24]. Treatment was administered 4 days before ischaemia (time required to induce Treg-cell proliferation, data not shown). We analysed the percentage of change of infarct volume at days 3, 7 and 10 with respect to the initial infarct volume and observed a significant reduction in infarct volumes at day 10 between controls (−22.12 ± 2.91%) and CD28-SA-treated animals (*versus* −33.37 ± 4.14%, *P* = 0.043). We also observed that oedema was significantly lower at day 7 in CD28-SA-treated animals (0.95 ± 0.33% *versus* 2.8 ± 0.35%, *P* = 0.002; Fig. 5).

### Treg cells' protection lasts for longer periods

**Fig. 6:**
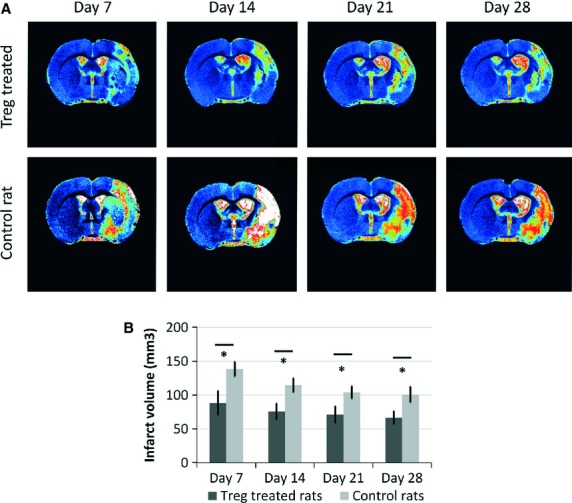
Temporal profiles of infarct sizes and oedema in control and Treg-treated animals, as measured by MRI. (**A**) T2 weighted MR images showing the temporal evolution of the lesion for control and treated animals. (**B**) Infarct volume measured for control (*n* = 8) and Treg-treated rats (*n* = 8) at days 7, 14, 21 and 28 post-MCA occlusion. Columns represent mean values, and error bars represent SEM (* indicates statistically significant differences; *P* < 0.05).

The temporal evolution of lesion sizes for control and Treg-treated animals is presented in Figure 6A. Treg-treated animals presented smaller brain infarcts compared with controls at day 7 (88.42 ± 17.63 *versus* 138.56 ± 10.92 mm^2^; *P* = 0.026), day 14 (75.68 ± 11.66 *versus* 114.62 ± 10.17 mm^2^; *P* = 0.023), day 21 (71.13 ± 12.21 *versus* 103.99 ± 9.11 mm^2^; *P* = 0.045) and day 28 (66.61 ± 9.48 *versus* 100.87 ± 11.64 mm^2^; *P* = 0.046) after occlusion (Fig. 6B).

### Treg cells did not impair endogenous neuroblasts division or angiogenesis

**Fig. 7:**
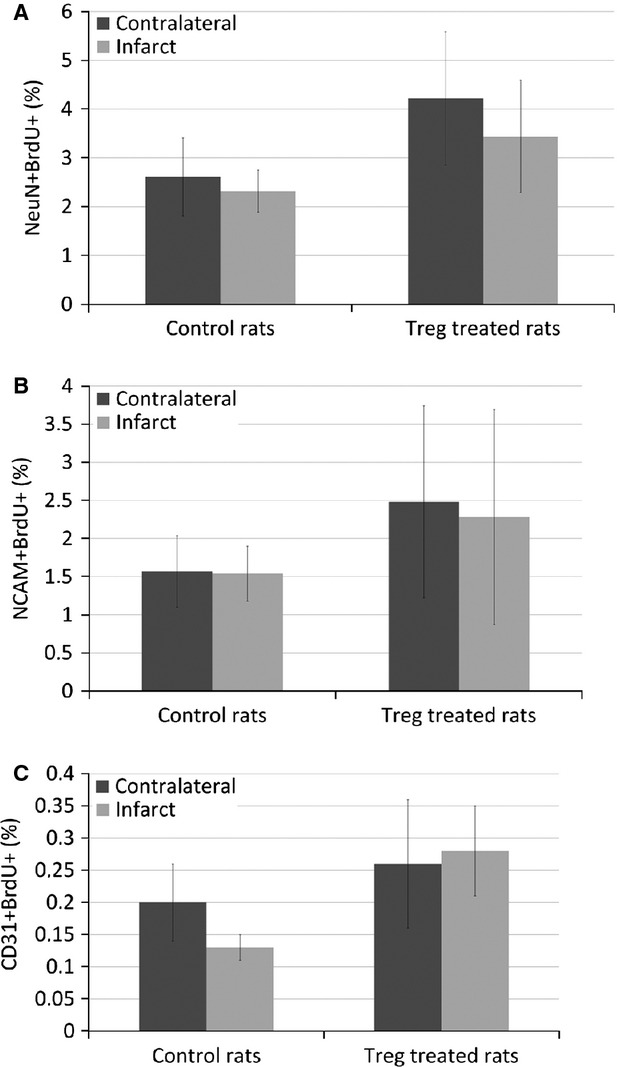
Measurement of neuroblast division and endothelial cells division by flow cytometry. Quantification of NeuN+BrdU+ cells (**A**), NCAM+BrdU+ cells (**B**) and CD31+BrdU+ cells (**C**) in the contralateral and ipsilateral hemispheres of Treg-treated (*n* = 8) and control rats (*n* = 8).

We analysed the percentage of NeuN+BrdU+ and NCAM+BrdU+ cells to quantify neural proliferation, and the percentage of CD31+BrdU+ cells to assess angiogenesis. The treatment with Treg cells did not impair endogenous cell proliferation in any case (Fig. 7). The slightly higher proliferation that resulted in the Treg-treated group was not statistically significant.

## Discussion

Stroke triggers an intense inflammatory response predominately mediated by the accumulation of inflammatory cells and mediators in the ischaemic brain. In this context, at late stages of the disease, Treg cells are activated, and they may participate in limiting the inflammatory response.

In this study, we demonstrated that Treg cells reduce the infarct volume in rats subjected to transient brain ischaemia. Animals treated with Treg cells, 2 hrs after occlusion of the MCA, showed a 35.5% reduction in infarct volume from day 3 up to day 28. In addition, Treg cells reduced brain oedema by 35.7% at day 3. Infarct volumes were also reduced when animals were treated with CD28-SA, an inductor of endogenous Treg proliferation. Overall, we believe that these findings suggest a neuroprotective effect of the Treg cells.

Previous studies reported an increase in FoxP3 expression and in Treg-cell numbers in the spleen and blood following experimental ischaemia [10] and differences in Treg-cell numbers in the blood from patients 7 days after a stroke [3]. In addition, several studies have attempted to reveal the role of Treg cells in animal models, by depleting these cells before the induction of ischaemia. However, the results of those studies are controversial. Liesz *et al*. [13] and Kleinschnitz *et al*. [15] showed that Treg cells exacerbated brain injury early after transient ischaemia, whereas Ren *et al*. [14] and Li *et al*. [16] described that T-cell therapy protected against cerebral ischaemia [15,16]. The discrepancies in these results could be derived from the different methods that were used in these studies. Thus, new studies are necessary to evaluate the potential therapeutic usefulness of Treg cells in brain ischaemia.

In this study, we aimed to investigate whether Treg cells exert protection following stroke. In our strategy, instead of depleting Treg cells, we aimed to analyse if the adoptive transfer of Treg cells or the stimulation of endogenous Treg cells could be a therapeutic strategy for the treatment of stroke. Both strategies reduced the infarct size from day 3 after stroke and, in our opinion, these strategies are more translational to the clinic. According to the STAIR criteria [26,27], we prolonged the observation periods after ischaemia, observing that protection is also maintained at days 14, 21 and 28.

We tried to investigate the mechanisms of Treg-cell protections; thus, we evaluated inflammation and, at longer time-points, neuroblasts division and angiogenesis. Higher levels of FoxP3 (Treg marker), lower levels of IL-1β and lower numbers of CD11b+ and CD68+ cells were found in the infarcted hemisphere of Treg-treated animals. These findings may indicate that Treg numbers in the brain are increased after stroke, and the protection observed could be mediated by a reduction in inflammation. At longer time-points (28 days), Treg-cell treatment did not impair the proliferation of NeuN+, NCAM+ or CD31+ cells, although a role in neuroblasts division and angiogenesis was not appreciated. Previous results in this regard were controversial, showing that Treg cells impair [17] or improve [18] angiogenesis after limb ischaemia. In our study, we showed that Treg cells did not impair neuroblasts division or angiogenesis.

In summary, we have found that the treatment of animals with Treg cells 2 hrs after experimental ischaemia reduces infarct volume from days 3 to 28. This reduction in infarct volume is accompanied by a higher expression of FoxP3 (Treg marker) and a reduction in inflammation in the infarcted brain hemisphere. These results suggest that adoptive transfer of Treg cells is a promising therapeutic strategy against the neurological consequences of stroke. Further studies should help elucidate the exact mechanism underlying this neuroprotective effect of Treg cells.
